# Progress of photodynamic therapy applications in the treatment of musculoskeletal sarcoma (Review)

**DOI:** 10.3892/ol.2014.2332

**Published:** 2014-07-09

**Authors:** XIANGHONG ZHANG, TANG LIU, ZHIHONG LI, XIANGSHENG ZHANG

**Affiliations:** Department of Orthopedics, The Second Xiangya Hospital, Central South University, Changsha, Hunan 410011, P.R. China

**Keywords:** photodynamic therapy, photodynamic diagnosis, sarcoma, musculoskeletal

## Abstract

Photodynamic therapy (PDT) has clinical approval for use as a minimally invasive therapeutic procedure that is able to exert selective cytotoxic activity toward pathological cells, particularly malignant cells. Following a number of recent technological improvements, PDT has been widely applied to the diagnosis and treatment of malignancies, including lung, esophageal, gastrointestinal, bladder, prostate, head and neck, oral and skin cancer. Studies have shown that osteosarcoma is a malignant tumor afflicting young adults worldwide, and recently, the incidence of bone and soft-tissue malignant tumors has been shown to be increasing, so the use of PDT has become an area of focus for the diagnosis and treatment of musculoskeletal sarcoma.

## 1. History and fundamentals of PDT

Photodynamic therapy (PDT), which is based on the dye-sensitized photooxidation of biological matter in the target tissue (1), has clinical approval for use as a minimally invasive therapeutic procedure that is able to exert selective cytotoxic activity toward pathological cells, particularly malignant cells. PDT has the potential to fulfill a number of currently unmet medical requirements. As a consequence, PDT has received increasing levels of attention since regulatory approval was granted to several photosensitizing drugs (2).

### History

A medical student named Raab unintentionally discovered that paramecia incubated in a fluorescent dye were destroyed once light was introduced (3). Raab’s professors, Jesionek and von Tappeiner, further analyzed this novel light-based therapy, resulting in the discovery of PDT. The photodynamic effect was then widely studied in cells, animals and the clinic. Despite this early success, this novel therapy did not reach a wide audience and was lost for nearly 50 years. Dougherty (4) accidentally rediscovered PDT and brought this novel therapy to the attention of a worldwide audience in the late 1970s and early 1980s. Based on the previous research, Dougherty developed commercially suitable photosensitizers (PSs) and reliable light sources, and performed appropriate clinical trials to demonstrate the value of PDT to the oncological community. Although a number of other important individuals contributed to the development of PDT, Dougherty is referred to as ‘the Father of PDT’ for making an outstanding contribution in bringing the therapy to light globally. Good results have subsequently been yielded from PDT, mainly in early-stage epithelial cancers, including lung (5), esophageal (6), gastrointestinal (7), bladder (8), prostate (9), head and neck (10), oral (11) and skin (12) cancer.

### Fundamentals of PDT

The fundamentals of PDT are based on an old concept that inert material can be transformed to active material following irradiation with light. The components of PDT must be analyzed and defined in order to achieve an improved understanding of the mechanism of PDT. Fundamentally, a PS is introduced and activated by light, which, in the presence of oxygen, may create a photodynamic reaction (PDR) (13).

The first step in PDT is the delivery of the PS to the target tissue. Throughout the delivery process, the photophysical, photochemical and biological characteristics of PSs should be maintained. The ideal result is for the PS to accumulate only in the target tissue. Previous studies have shown that a number of factors contribute to this. For example, Matsubara (14) found that acridine orange (AO) used in PDT accumulates in malignant musculoskeletal tumors depending on the pH gradient. The second step in PDT is to bring PS-activating light to the target tissue. It has been shown that for successful activation of each PS, a unique wavelength of light and intensity of light fluence is required (13). A PDR may then occur upon exposure of the specific PS to light of the appropriate wavelength and intensity, ideally allowing for tumor damage without undue destruction of normal tissues (15).

When bathed in light, the PS changes to an extremely unstable state and also transfers energy to form molecular oxygen. A significant and complex cascade of events then occurs, resulting in local, regional and systemic alterations and tumor and immune responses. Tumor destruction from PDT can occur by cellular effects, vascular effects or both. Cellular lethality may be caused by an imbalance in the mitochondria, lysosomes, plasma, hydrolytic enzymes, certain cytokines and calcium, or even by DNA damage. As a result, the tumor cells are eliminated through the programmed (apoptotic) and non-programmed (necrosis) cell death pathways (16,17). Generally, studies have shown that the type of cell death is dependent on light intensity, with rapid death by necrosis using a high light intensity, and PDT initiating apoptotic death under low light doses (18–20). Similar to results found in tumors, studies have also shown that the PS in the vasculature may also create a PDR when activated by the appropriate light parameters (21,22). Gross edema and erythema are always the first signs of a PDT response, particularly where skin is involved in the treatment field (23). Eventual vessel occlusion also appears to be a general phenomenon accompanying PDT. Overall, vessel occlusion leads to lethality of the tumor by a rapid loss of blood supply and vascular cell lysis.

Additionally, pre-clinical and clinical studies have shown that PDT treatment of a tumor can enhance systemic anti-tumor immunity and surveillance (24).

## 2. Factors that play significant roles in PDT

### PSs

PSs plays a significant role in PDT, as the agents are natural or synthetic structures that transfer light energy (25). While a large number of PSs have been tested in *vivo* and *in vitro* in PDT experiments, to date, there have been few that have shown ideal properties (26). Since the identification of the first PS and its use in the clinic, novel PSs have regularly been identified and reported. Although clinicians and chemists hold different views on which is the ideal PS (25), they are in agreement on the characteristics that an optimal PS should possess, for clinical PDT at least.

Firstly, the PS should be commercially available to a worldwide audience, so that all suitable cancer patients can benefit from PDT and undergo this therapy. Secondly, the drug should have chemical purity and be easily shipped and transported in a stable state. It has been shown that the PS requires hydrophilicity to travel safely and lipophilicity to bind appropriately to the target; this combination is termed amphilicity (27). Thirdly, the ideal PS should also have the characteristics of chemical and physical stability, good selectivity towards target cells, low dark toxicity, but strong photocytotoxicity, activation at a long wavelength and rapid removal from the body. A previous study showed that agents accumulated not only in cancer cells, but also in normal cells, and that light irradiation of normal cells would result in their death (28). Fourthly, reliable and pain free activation of the PS are of significant clinical benefit, as few patients are willing to repeat a painful experience even if it is beneficial to their health. As DNA damage is less in PDT-treated cells compared with those irradiated by other means, it is a great benefit that PDT does not appear to be carcinogenic when compared with chemotherapy or radiation therapy. So, fifthly, the PS should allow the PDT to be integrative and complimentary to other oncological interventions, including surgery, chemotherapy and radiation therapy, rather than preventing these treatments and therefore becoming viewed as competitive in nature (27).

Recently, more PSs have been discovered and attempts have been made to classify them. Although PSs can be categorized in various ways, all methods have their limitations. Certain scholars have tried to separate the PSs based on when they were generated. Traditionally, porphyrin-based and included hematoporphyrin and its derivatives or synthetics are termed first generation PSs. The PS of this generation are usually mixtures and have a number of drawbacks, including poor selectivity and stability, slow clearance, a long retention time in normal tissues and the easy induction of photoallergic reactions. Therefore, in order to overcome these supposed deficiencies, the second generation PSs, including 5-aminolaevulinic acid (5-ALA) (29), AO and benzoporphyrin (30), were developed in the late 1980s. The third generation PSs generally refer to the modifications of the first and second generations and include molecular conjugates, antibody conjugates, liposome conjugates and nanoparticles (31). At present, this generation of PSs is in the experimental research stage. For each generation, in order to deliver the PS exactly to the neoplasia or receptors expressed on specific tumors, the PS requires attachment to various molecules, such as monoclonal antibodies, antibody fragments, peptides, proteins and various carbohydrates (32).

### Light sources

The light source is also a significant factor in PDT (33). Sunlight, having the intensity and diversity of wavelength to activate a number of PSs, was the first light source used in PDT. While sunlight is easy to use with low costs, it has a number of disadvantages, including the thermal effect, shallower tissue penetration, a low light intensity and difficulty in controlling the dose of spatial and temporal distribution (28). Generally, in order to perform PDT successfully, it is necessary to ensure that sufficient light reaches all the diseased tissues and to active more PSs (34).

There are numerous factors affecting the manner in which light travels through various tissues and correctly reaches the target molecule. It has been demonstrated that different spectral light sources may be absorbed by different biological tissues due to endogenous tissue chromophores, such as hemoglobin, myoglobin and cytochromes (35). Also, different PSs have different maximum absorption wavelength ranges. Therefore, in order to produce a suitable cytotoxic effect, the spectral characteristics of the light must coincide with the wavelength range of the PS. The energy dose of the light and the excitation time are also factors that should be considered (32). Satonaka *et al* (36) demonstrated that flash-wave light (FWL) strongly enhanced the cytocidal effect of PDT with AO in a mouse osteosarcoma cell line.

Due to developments in optical physics, the majority of these drawbacks can be overcome by carefully designed light sources, including the xenon lamp, light emitting diode (LED) (31), laser beam and fiber optic devices. Each method of illumination has its own advantages and disadvantages. The xenon lamp can illuminate a wide tumor area in one burst and the illuminated range can be easily changed; unfiltered xenon light has a peak spectrum in the wavelength range of 450 to 550 nm (36). The xenon lamp is also much cheaper than a laser (37). A laser beam, while possessing a high energy output, can only illuminate a narrow tumor area. Additionally, a laser beam does not have a wide wavelength spectrum. LEDs and fiber optic devices are also used in PDT to treat clinical disease, although they are not widely used. Through continuing technological development, in the future, light sources should even be available using fluorescing agents and nanoparticles. This development will provide the advantages of minimizing the dose of PS and light to treat non-cutaneous lesions, as the light source will be able to interact directly with the tumor bed and potentially be in extremely close contact with the PS that is accumulated in the tumor. Therefore, the choice of an optimal combination of PS, light source and treatment parameters is crucial for successful PDT (32).

### Drug light interval (DLI)

The DLI is the interval time from the introduction of the PS to the illumination by the light source. The DLI also plays a significant role in controlling the PDT (38). The PS begins to travel from its vascular origin to the rest of the vasculature after it has been first introduced. As time passes, the PS is likely to accumulate in the tumor and blood. By early illumination, after the entrance of the PS, the vascular effects may predominate, i.e., a rapid DIL would favor vascular collapse. Tumor cell effects would be favored by later illumination. Therefore, potentially, a more selective tumor destroying effect could be provided by a prolonged DIL (39), whereas a rapid DLI would be the ideal option for a highly vascular tumor (40). Overall, the DLI should coincide with the type of tumor, PS, light source and entrance method of the PS; i.e., the DLI should be determined following careful consideration of the combination of these factors.

## 3. Application of PDT in the diagnosis and treatment of bone and soft-tissue malignant tumors

Infiltrating growth is the major pathological feature of primary malignant tumors of the bone and soft tissue. The incidence of these tumors is ~1% of all malignant tumors. Osteosarcoma is a malignant tumor afflicting young adults worldwide. A previous study reported that osteosarcoma is the most common malignant bone tumor, affecting ~400 children and adolescents annually in the United States (41). Recently, the incidence of bone and soft-tissue malignant tumors has been shown to be increasing. The tumors are highly malignant, with a high rate of metastasis, a poor prognosis and a high fatality rate; all factors that make treatment in the clinic difficult. The major therapeutic methods include operative treatment, radiotherapy, chemotherapy, immunotherapy, gene therapy and certain other innovative therapeutic methods, such as PDT and ultrasound radiation (42). PDT is becoming more widely applied for the diagnosis and treatment of malignant tumors. Recent experimental studies (43,44) have also revealed that PDT is a novel effective approach for treating malignancies such as osteosarcoma, Ewing’s sarcoma of the soft tissues and synovial sarcoma (SS).

Detection at an early, pre-malignant or intra-operative stage may increase the number of therapeutic options available and improve the quality of life and chances of survival for the patient. Various imaging methods are employed for the extracorporeal detection of malignant tumors in the human body, including scintigraphy X-ray, ultrasound, magnetic resonance imaging (MRI), computed tomography and other tomographic techniques. With regard to the surgical margin, identification of an apparent tumor margin is possible by clinical judgment and interpretation of the MRI findings, however, these results are evidently subjective. If MRI could detect a single or small number of tumor cells, the resection of tumors with a safe or adequate surgical margin would be easy. A number of these methods are time consuming and subject to sampling error, as the majority of dysplastic areas are not visibly distinct (45). Therefore, at present, it is recommended that surgeons decide on the surgical margins by relying on their experience and based on MRI or other imaging methods that are currently unable to detect single cells. Compared with the aforementioned techniques, photodynamic diagnosis (PDD) provides an innovative, non-invasive method for the detection of a single cell and is a safer imaging technology (31). Stübel (46) first recognized the potential of tissue fluorescence for diagnostic purposes in 1911. PSs, taking AO as an example, have two characteristics: One is the apparently selective accumulation in tumors, and the other is the ability to emit fluorescence when excited by specific light. These two features allow direct visualization of AO accumulation in tumors and is termed the fluorovisualization effect (47).

According to previous studies, surgery is the most important and widely used method for treating malignant bone tumors. Surgery should strive to resect the lesion thoroughly so as to avoid recurrence, while retaining limb function, therefore, the surgeries are contradictory. As it would be preferable to be able to distinguish between tumor cells and normal cells during surgery, Kusuzaki *et al* (48) studied this issue and found that the fluorovisualization effect is extremely useful for detecting tumor tissues during surgery. In the study, 32 patients with musculoskeletal sarcoma lesions were treated; the resultant figures represented a recurrence rate of 25%, which is an acceptable result for lesional recurrence, compared with conventional surgery. The study indicated that residual tumor tissues even <1 mm in diameter can be detected and excised following tumor excision with an intra-lesional or minimal margin. Kusuzaki *et al* (49) also showed that local tumor recurrence was significantly inhibited (23%) in a group treated with curettage under fluorovisualization and AO-PDT, compared with the control group (80%) treated with curettage alone under ordinary light. Since PDD has the ability to make a distinction between normal and degenerative cells, fixation of the appropriate dose and time is an issue that requires immediate attention. Satonaka *et al* (50) reported the results of a study investigating the feasibility of PDD utilizing the fluorovisualization effect of AO for the extracorporeal detection of mouse osteosarcoma inoculated into the soft tissues. It was revealed that the optimal condition for clear detection of the osteosarcoma was evaluation 2 h after the intravenous injection of AO at 0.1 mg/kg. However, different PSs and different types of bone and soft-tissue malignant tumors may have varying appropriate doses and DLIs. Therefore, in order for the best usage of PDD in bone and soft-tissue tumors, it is necessary to consider all factors, which is consistent with the aforementioned principal elements. The manner in which the PSs of PDD are transported includes using local, intravenous and intratracheal pathways. Studies have shown that the systemic delivery of AO via intravenous administration may be useful for the PDD of mouse osteosarcoma; this method can homogeneously expose all tumor cells without complications (50,51). Although at present there is no clear and convincing evidence with regard to which pathway is the most effective for transportation, it is an area of interest that is worthy of further study. Overall, PDD using PSs, such as AO, ALA (31) and methylene blue (52), may be feasible for the detection of human musculoskeletal sarcomas, including the use of pre-operative and intraoperative diagnosis, which also aids in the complete excision of lesions. PDD will play a significant role in the future clinical diagnosis of bone and soft-tissue tumors.

With regard to treatment, as studies have shown that tumors exhibit unusual proliferation and differentiation and abnormal apoptosis, the enhancement of apoptosis is an important mechanism behind numerous therapeutic modalities (53,54), including PDT, which can usually be performed as a day-case procedure without the requirement for general anesthesia or strong analgesia. PDT also tends to be a more cost-effective treatment option. There are consequently a number of innovative studies on the use of PDT in the treatment of bone and soft-tissue malignant tumors.

Prior to 1980, bone and soft-tissue malignant sarcomas of the extremities were treated almost exclusively by amputation in order to achieve adequate margins to control the disease (55). While amputation has significant morbidity due to loss of function and disfigurement, wide local excision exhibits a greater risk of local recurrence. AO (56), which specifically binds to malignant tumors and immediately accumulates in tumor cells (57), is a promising novel PS for treating musculoskeletal sarcoma. Therefore AO-PDT has been suggested to be an innovative therapeutic method for musculoskeletal sarcoma patients and has been used since 1999. Limb-salvage surgery aims to balance adequate excision margins for disease control and the preservation of all important structures in order to retain maximum functionality (58). Recovery of limb function subsequent to limb salvage surgery with a wide tumor resection, followed by limb reconstruction, has not yet been satisfactorily achieved, therefore, there is a requirement to explore novel methods for achieving this. In order to investigate the rate of recovery, Kusuzaki *et al* (49) performed a clinical trial using ten patients, including four patients with SS and single patients with osteosarcoma, chondrosarcoma, Ewing’s sarcoma, rhabdomyosarcoma, periosteal osteosarcoma, alveolar soft part sarcoma and primary musculoskeletal sarcoma, respectively, in order to investigate whether AO-PDT is more useful than other conventional methods. The results showed that PDT achieved a local recurrence rate of <10%, excellent limb function and a low metastasis rate.

AO is not the only good choice of PS, as certain others, including ALA, can generate different results. Further types, including antibody conjugate, liposome conjugate and nanoparticles are also good choices. Numerous other studies have clarified the same idea, and further experiments have been performed, such as those in the study by Ueda *et al* (59). Ueda *et al* (59) demonstrated that PDT with AO exerts a rapid cytocidal effect on mouse osteosarcoma *in vitro* and *in vivo*. In their experiment, in order to clarify which was the appropriate lesion and which was affected by AO-PDT clinically, the mouse osteosarcoma cell line was selected. with different concentrations and exposure times as the variables. Unfiltered xenon light was determined as the light source for AO-PDT, and PDT was shown to enhance the cytocidal effect of AO-PDT in the mouse osteosarcoma cells. As it has been shown that chemosensitive cells are sensitive to PDT, studies have also been performed to identify whether PDT would also be a good choice for multidrug-resistant tumors. Kusuzaki *et al (*60) discovered that AO-PDT has a strong cytocidal effect, not only on chemosensitive mouse osteosarcoma cells, but also on multidrug-resistant mouse osteosarcoma cells.

Osteosarcoma is the most common primary malignant bone tumor, and studies have shown that the lung is the most common target for osteosarcoma metastasis (61). Asai *et al* (62) reported that pulmonary metastasis occurs in 40–50% of patients with osteosarcoma and remains a major cause of a fatal outcome. Studies by Rasalkar *et al* (41) showed that the overall survival rate among patients with localized osteosarcoma without metastatic disease is 60–70%, whereas the survival rate reduces to 10–30% in patients with metastatic disease (63). Metastasis has conferred numerous disadvantages on the treatment of osteosarcoma. Therefore, in order to avoid these difficulties, certain studies have indicated that PDT is the best choice of treatment. When working on LM8, which possesses strong metastatic ability, Satonaka *et al* (63) demonstrated that AO-PDT, using FWL as the excitation light following intravenous AO injection, was highly effective for inhibiting the growth of pulmonary metastases from mouse primary osteosarcomas. It was also suggested that AO-PDT would prevent primary osteosarcoma metastasis to the lung. However, we do not yet understand the mechanisms of this function.

PDT is also sensitive and useful in the diagnosis and treatment of SS, Ewing’s sarcomas, malignant fibrous histiocytomas (49) and other bone and soft-tissue malignant tumors. SS frequently involves or invades the major neurovascular structures and bones, and its local recurrence rate following simple resection has been reported to be as high as 80% (64). Kusuzaki *et al* (64) performed a clinical trial with six cases of SS to analyze the use of AO-PDT, and concluded that AO-PDT with 5 Gy irradiation may be an excellent novel therapeutic modality, together with reduction surgery, to salvage limb function in SS involving major nerves and vessels or bones. Through a study of a rare case of periosteal Ewing’s sarcoma, Yoshida *et al* (65) also suggested that reduction surgery with PDT may be the strategy of choice to obtain satisfactory limb function in cases of periosteal Ewing’s sarcoma.

The use of PDT combined with other therapies, such as metronomic or nanoparticle PDT, may also lead to the advancement of PDT. For example, there are several additional advantages of AO therapy combined with electronic magnetic hyperthermia treatment (EMHT): i) Visualization of the tumor cell by fluorescent microscopy during the surgery; ii) removal of the visualized target cell without damage to healthy tissues; and iii) residual AO-labeled tumor cells seated in deep areas are killed by low-dose radiation following surgery. With these advantages, the combination of AO therapy and EMHT has the ability to replace conventional tumor surgery and radiotherapy in certain cases (66). Therefore, it may strongly be suggested that PDT can be widely used in the diagnosis and treatment of bone and soft-tissue malignant tumors in the future.

## 4. Summary

The survival rate for bone and soft-tissue malignant tumors remains low, although there has recently been great progress in their diagnosis and treatment, in the use of salvage surgery with various types of endoprostheses, and in the wide development of bone allografts or autografts for the treatment of musculoskeletal sarcomas. Recent experimental studies have revealed that PDT is a novel effective approach for treating bone and soft-tissue malignancies. However, the poor penetration of light through biological tissues is a major limitation of the clinical application of PDT, and the ideal PS and light source require further in-depth research. However, PDT remains a novel and promising anti-tumor strategy. Compared with surgery, chemotherapy or radiotherapy, PDT has the advantages of reducing long-term morbidity, low-compromising to future disease, a good efficacy for repeated usage and carries no risk to immunity. To overcome the shortfalls of PDT in the treatment of bone and soft-tissue malignant tumors, improvements could be made by finding an ideal PS and light source. PDT combined with other therapies, such as metronomic or nanoparticle PDT, would also aid the advancement of PDT. Overall, the development of PDT to diagnose and treat bone and soft-tissue malignant tumors should progress rapidly with the identification of a novel ideal PS and light source, and PDT is likely to become a routine treatment for tumors in the future.

## References

[1] Sharma SK, Mroz P, Dai T, Huang YY, St Denis TG, Hambin MR (2012). Photodynamic therapy for cancer and for infections: What is the difference?. Isr J Chem.

[2] Brown SB, Brown EA, Walker I (2004). The present and future role of photodynamic therapy in cancer treatment. Lancet Oncol.

[3] Allison RR, Mota HC, Sibata CH (2004). Clinical PD/PDT in North America: an historical review. Photodiagnosis Photodyn Ther.

[4] Dougherty TJ (1984). Photodynamic therapy (PDT) of malignant tumors. Crit Rev Oncol Hematol.

[5] Hayata Y, Kato H, Konaka C (1984). Photoradiation therapy with hematoporphyrin derivative in early and stage 1 lung cancer. Chest.

[6] McCaughan JS, Ellison EC, Guy JT (1996). Photodynamic therapy for esophageal malignancy: a prospective twelve-year study. Ann Thorac Surg.

[7] Cheung J, Todd M, Turnbull R (2004). Longer term assessment of photodynamic therapy for Intimal Hyperplasia: a pilot Study. J Photochem Photobiol B.

[8] Cheung G, Sahai A, Billia M, Dasgupta P, Khan MS (2013). Recent advances in the diagnosis and treatment of bladder cancer. BMC Med.

[9] Bhowmick R, Girotti AW (2014). Pro-survival and pro-growth effects of stress-induced nitric oxide in a prostate cancer photodynamic therapy model. Cancer Lett.

[10] Hwang H, Biswas R, Chung PS, Ahn JC (2013). Modulation of EGFR and ROS induced cytochrome *c* release by combination of photodynamic therapy and carboplatin in human cultured head and neck cancer cells and tumor xenograft in nude mice. J Photochem Photobiol B.

[11] Rigual N, Shafirstein G, Cooper MT (2013). Photodynamic therapy with 3-(1′-Hexyloxyethyl) pyropheophorbide a for cancer of the oral cavity. Clin Cancer Res.

[12] Shi L, Wang X, Zhao F (2013). In vitro evaluation of 5-aminolevulinic acid (ALA) loaded PLGA nanoparticles. Int J Nanomedicine.

[13] Allison RR, Moghissi K (2013). Photodynamic therapy (PDT): PDT mechanisms. Clin Endosc.

[14] Matsubara T, Kusuzaki K, Matsumine A, Shintani K, Satonaka H, Uchida A (2006). Acridine orange used for photodynamic therapy accumulates in malignant musculoskeletal tumors depending on pH gradient. Anticancer Res.

[15] Sibata CH, Colussi VC, Oleinick NL, Kinsella TJ (2001). Photodynamic therapy in oncology. Expert Opin Pharmacother.

[16] Oleinick NL, Morris RL, Belichenko I (2002). The role of apoptosis in response to photodynamic therapy: what, where, why, and how. Photochem Photobiol Sci.

[17] Igney FH, Krammer PH (2002). Death and anti-death: tumour resistance to apoptosis. Nat Rev Cancer.

[18] Oleinick NL, Evans HH (1998). The photobiology of photodynamic therapy: cellular targets and mechanisms. Radiat Res.

[19] Ding X, Xu Q, Liu F (2004). Hematoporphyrin monomethyl ether photodynamic damage on HeLa cells by means of reactive oxygen species production and cytosolic free calcium concentration elevation. Cancer Lett.

[20] Henderson BW, Donovan JM (1989). Release of prostaglandin E2 from cells by photodynamic treatment in vitro. Cancer Res.

[21] Castano AP, Demidova TN, Hamblin MR (2005). Mechanisms in photodynamic therapy: part three - photosensitizer pharmacokinetics, biodistribution, tumor localization and modes of tumor destruction. Photodiagnosis Photodyn Ther.

[22] Castano AP, Demidova TN, Hamblin MR (2005). Mechanisms in photodynamic therapy: part two - cellular signaling, cell metabolism and modes of cell death. Photodiagnosis Photodyn Ther.

[23] Henderson BW, Dougherty TJ (1992). Review article: How does photodynamic therapy work?. Photochem Photobiol.

[24] Kurohane K, Tominaga A, Sato K, North JR, Namba Y, Oku N (2001). Photodynamic therapy targeted to tumor-induced angiogenic vessels. Cancer Lett.

[25] Allison RR, Downie GH, Cuenca R, Hu XH, Childs CJ, Sibata CH (2004). Photosensitizers in clinical PDT. Photodiagnosis Photodyn Ther.

[26] De Rosa FS, Bentley MV (2000). Photodynamic therapy of skin cancers: sensitizers, clinical studies and future directives. Pharm Res.

[27] Allison RR, Sibata CH (2010). Oncologic photodynamic therapy photosensitizers: a clinical review. Photodiagnosis Photodyn Ther.

[28] Vrouenraets MB, Visser GW, Snow GB, van Dongen GA (2003). Basic principles, applications in oncology and improved selectivity of photodynamic therapy. Anticancer Res.

[29] Kennedy JC, Pottier RH (1992). Endogenous protoporphyin IX, a clinically useful photosensitizer for photodynamic therapy. J Photochem Photobiol B.

[30] Burch S, Boqaards A, Siewerdsen J (2005). Photodynamic therapy for the treatment of metastatic lesions in bone: studies in rat and porcine models. J Biomed Opt.

[31] Huang Z (2005). A review of progress in clinical photodynamic therapy. Technol Cancer Res Treat.

[32] Agostinis P, Berg K, Cengel KA, Foster TH, Girotti AW (2011). Photodynamic therapy of cancer: an update. CA Cancer J Clin.

[33] Castano AP, Demidova TN, Hamblin MR (2004). Mechanisms in photodynamic therapy: part one - photosensitizers, photochemistry and cellular localization. Photodiagnosis Photodyn Ther.

[34] Ochsner M (1996). Light scattering of human skin: a comparison between zinc (II)-phthalocyanine and photofrin II. J Photobiol B.

[35] Robertson CA, Evans DH, Abrahamse H (2009). Photodynamic therapy (PDT): a short review on cellular mechanisms and cancer research applications for PDT. J Photochem Photobiol B.

[36] Satonaka H, Kusuzaki K, Matsubara T, Shintani K, Wakabayashi T, Nakamura T (2007). Flash wave light strongly enhanced the cytocidal effect of photodynamic therapy with acridine orange on a mouse osteosarcoma cell line. Anticancer Res.

[37] Alexiades-Armenakas M (2006). Laser-mediated photodynamic therapy. Clin Dermatol.

[38] Hamblin MR, Rajadhyaksha M, Momma T, Soukos NS, Hasan T (1999). In vivo fluorescence imaging of the transport of charged chlorin e6 conjugates in a rat orthotopic prostate tumour. Br J Cancer.

[39] Kurohane K, Tominaga A, Sato K, North JR, Namba Y, Oku N (2001). Photodynamic therapy targeted to tumor-induced angiogenic vessels. Cancer Lett.

[40] Seshadri M, Spernyak JA, Mazurchuk R, Camacho SH, Oseroff AR, Cheney RT, Bellnier DA (2005). Tumor vascular response to photodynamic therapy and the antivascular agent 5,6-dimethylxanthenone-4-acetic acid: implications for combination therapy. Clin Cancer Res.

[41] Rasalkar DD, Chu WC, Lee V, Paunipagar BK, Cheng FW, Li CK (2011). Pulmonary metastases in children with osteosarcoma: characteristics and impact on patient survival. Pediatr Radiol.

[42] Tian Z, Quan X, Xu C, Dan L, Guo H, Leung W (2009). Hematoporphyrin monomethyl ether enhances the killing action of ultrasound on osteosarcoma in vivo. J Ultrasound Med.

[43] Nomura L, Yanase S, Matsumura Y, Nagai K, Tagawa T (2004). Efficacy of combined photodynamic and hyperthermic therapy with a new light source in an in vivo osteosarcoma tumor model. J Clin Laser Med Surg.

[44] Hashiguchi S, Kusuzaki K, Murata H (2002). Acridine orange excited by low-dose radiation has a strong cytocidal effect on mouse osteosarcoma. Oncology.

[45] Messmann H, Knuchel R, Baumler W (1999). Endoscopic fluorescence detection of dysplasia in patients with Barrett’s esophagus, ulcerative colitis, or adenomatous polyps after 5-aminolevulinic acid-induced protoporphyrin IX sensitization. Gastrointest Endosc.

[46] Stübel H (1911). The fluorescence of animal tissues in ultraviolet light. Pflüger’s Archiv für die gesamte Physiologie des Menschen und der Tiere.

[47] Kusuzaki K, Suginoshita T, Minami G (2000). Fluorovisualization effect of acridine orange on mouse osteosarcoma. Anticancer Res.

[48] Kusuzaki K, Aomori K, Suginoshita T (2000). Total tumor cell elimination with minimum damage to normal tissues in musculoskeletal sarcomas following photodynamic therapy with acridine orange. Oncology.

[49] Kusuzaki K, Murata H, Matsubara T, Satonaka H, Wakabayashi T, Matsumine A, Uchida A (2007). Review. Acridine orange could be an innovative anticancer agent under photon energy. In vivo.

[50] Satonaka H, Kusuzaki K, Matsubara T, Shintani K, Wakabayashi T, Matsumine A, Uchida A (2006). Extracorporeal photodynamic image detection of mouse osteosarcoma in soft tissues utilizing fluorovisualization effect of acridine orange. Oncology.

[51] Kusuzaki K, Murata H, Matsubara T (2005). Clinical trial of photodynamic therapy using acridine orange with/without low dose radiation as new limb salvage modality in musculoskeletal sarcomas. Anticancer Res.

[52] Matsubara T, Kusuzaki K, Matsumine A, Satonaka H, Shintani K, Nakamura T, Uchida A (2008). Methylene blue in place of acridine orange as a photosensitizer in photodynamic therapy of osteosarcoma. In Vivo.

[53] Hickman JA (2002). Apoptosis and tumourigenesis. Curr Opin Genet Dev.

[54] Hanahan D, Weinberg RA (2000). The hallmarks of cancer. Cell.

[55] Springfield DS (1991). Introduction to limb-salvage surgery for sarcomas. Orthop Clin North Am.

[56] Kusuzaki K, Aomori K, Suginoshita T (2000). Total tumor cell elimination with minimum damage to normal tissues in musculoskeletal sarcomas following photodynamic therapy with acridine orange. Oncology.

[57] Kusuzaki K, Murata H, Takeshita H (2000). Intracellular binding sites of acridine orange in living osteosarcoma cells. Anticancer Res.

[58] Wright EH, Gwilym S, Gibbons CL, Critchley P, Giele HP (2008). Functional and oncological outcomes after limb-salvage surgery for primary sarcomas of the upper limb. J Plast Reconstr Aesthet Surg.

[59] Ueda H, Murata H, Takeshita H, Minami G, Hashiguchi S, Kubo T (2005). Unfiltered xenon light is useful for photodynamic therapy with acridine orange. Anticancer Res.

[60] Kusuzaki K, Minami G, Takeshita H (2000). Photodynamic inactivation with acridine orange on a multidrug-resistant mouse osteosarcoma cell line. Jpn J Cancer Res.

[61] Wu PK, Chen WM, Chen CF, Lee OK, Haung CK, Chen TH (2009). Primary osteogenic sarcoma with pulmonary metastasis: clinical results and prognostic factors in 91 patients. Jpn J Clin Oncol.

[62] Asai T, Ueda T, Itoh K, Yoshioka K, Aoki Y, Mori S, Yoshikawa H (1998). Establishment and characterization of a murine osteosarcoma cell line (LM8) with high metastatic potential to the lung. Int J Cancer.

[63] Satonaka H, Kusuzaki K, Akeda K (2011). Acridine orange inhibits pulmonary metastasis of mouse osteosarcoma. Anticancer Res.

[64] Kusuzaki K, Murata H, Matsubara T (2005). Clinical outcome of a novel photodynamic therapy technique using acridine orange for synovial sarcomas. Photochem Photobiol.

[65] Yoshida K, Kusuzaki K, Matsubara T (2005). Periosteal Ewing’s sarcoma treated by photodynamic therapy with acridine orange. Oncol Rep.

[66] Matsubara T, Matsumine A, Kusuzaki K (2013). A minimally invasive surgery for bone metastases using the combination of photodynamic therapy and hyperthermia treatment. Inter J Clin Med.

